# The Association between Postoperative Cognitive Dysfunction and Cerebral Oximetry during Geriatric Orthopedic Surgery: A Randomized Controlled Study

**DOI:** 10.1155/2021/5733139

**Published:** 2021-10-19

**Authors:** Junqiang Zhu, Wei Wang, Huimin Shi

**Affiliations:** Department of Anesthesiology, No. 2 People's Hospital of Yuhang District, Hangzhou 311121, China

## Abstract

**Background:**

Postoperative cognitive dysfunction (POCD) refers to disorders affecting orientation, attention, perception, consciousness, and judgment that develop after geriatric orthopedic surgery. Cerebral blood oxygen saturation detection is a way to diagnose cerebral oxygen supply during operation. At present, more and more applications are used for early diagnosis of postoperative cognitive function. Therefore, the present study is to analyze the relationship between postoperative cognitive dysfunction and cerebral blood oxygen saturation in elderly orthopedic patients.

**Methods:**

This study enrolled 90 elderly patients undergoing orthopedic surgery in our hospital. According to the postoperative cognitive dysfunction, they were divided into POCD group (*N* = 45) and no-POCD (*N* = 45) group. The cognitive and psychological function and cerebral blood oxygen saturation were analyzed before and 3 months after the operation. Finally, the indicators of cognitive psychological function and the indicators of cerebral blood oxygen saturation are correlated and analyzed.

**Results:**

Compared with the normal group, patients with cognitive dysfunction at 3 months after surgery time below preoperative rScO2, time below a 10% decrease from preoperative rScO2, CDL preoperative, minimum rScO2 value, and maximum rScO2 value have significant changes. The results of the correlation analysis found that there is also a significant correlation between the postoperative cognitive and psychological function of the patient and the cerebral blood oxygen saturation at 3 weeks after the operation.

**Conclusion:**

In elderly orthopedic patients, there is a significant relationship between cerebral blood oxygen saturation detection and cognitive function 3 months after surgery.

## 1. Introduction

Postoperative cognitive dysfunction (POCD) refers to disorders affecting orientation, attention, perception, consciousness, and judgment that develop after surgery. The occurrence of postoperative cognitive dysfunction (POCD) was 30-80% in heart surgery and 10-60% in 3-6 months [1]. In addition, the incidence of cognitive dysfunction was 26% in patients undergoing noncardiac surgery [2]. POCD is associated with a variety of factors, such as age, level of well-asking, and mental health [3]. With the rapid arrival of the aging society, there are more and more complications in patients, which in turn leads to an increase in the incidence of complications and the risk of surgery. Short-term or long-term postoperative cognitive dysfunction (POCD) occurs after surgery in elderly patients [4]. This may be related to an increase in endotoxins during surgery, the formation of blood clots, and the release of endotoxins which mean that systemic inflammation plays important role in progress of POCD [5]. In addition, surgery is one of the most important reasons for POCD. The occurrence of POCD increases significantly as the time and length of surgery increase. This may be related to an increase in endotoxins during surgery and the formation of blood clots. The release of endotoxins stimulates the release of interletons and leads to systemic inflammation. In addition, the formation of hydrants, mainly fat hydrants, is an important cause of POCD [6, 7]. The prevention of PCOD mainly includes the detection of the depth of anesthesia and the depth of cerebral oxygenation during surgery, thus minimizing the damage of anesthesia [8].

Blood oxygen saturation detection is a noninvasive detection technology based on near-infrared spectroscopy, which has been obtained by the U.S. Food and Drug Administration. In heart-related diseases and surgery, it has been found that oxygen saturation testing in the brain region can be significantly related to the recovery of spontaneous circulation during CPR [9]. It has also been found in heart-related surgery that hypooxy saturation is often associated with cognitive states such as delirium after surgery [10, 11]. Similar patterns have been found in orthopedic-related surgeries, including applications in knee surgery and total hip replacement [12–16]. However, there is still a lack of relevant research on whether brain oxygen saturation monitoring during orthopedic surgery is related to cognitive function. Therefore, this study intends to detect brain saturation detection and then analyze the relationship between cognitive function and cerebral oxygenation in elderly patients after orthopedic surgery and the hypothesis of this study that there is a close relationship between cerebral oximetry and cognitive function.

## 2. Methods

### 2.1. Study Design

The study was conducted in a randomized controlled study from January 2019 to December 2019 and approved by the ethics committee of No. 2 People's Hospital of Yuhang District. We selected 90 patients who underwent joint surgery (total knee arthroplasty and total hip arthroplasty) and spinal surgery in our hospital and obtained written informed consent. The exclusion criteria of this study were that the preoperative minor mental status examination score was less than 24 points; the current and past history of mental illness or the history of central nervous system disease; the history of cardiovascular surgery or the history of craniotomy; the history of drug or alcohol dependence; history of liver failure or renal failure; and persons with a history of severe hearing and visual impairment.

### 2.2. Procedures

The brain oxygen saturation detection instrument used in this study is Narcotrend® monitor (Narcotrend®-Compact, MT MonitorTechnik GmbH und Co. KG, Germany) and near-infrared spectroscopy (INVOS 5100B, Somanetics, Troy, MI, USA). Three electrode pads and rSO2 sensors were connected to the skin of the forehead, and the values of Narcotrend and rSO 2 were recorded at intervals of 30 seconds. The process of induction of anesthesia is carried out by intravenous anesthesia, with continuous inhalation of sevoflurane, and intermittent infusion of vecuronium and fentanyl to maintain the anesthetic dose *μ*g/kg fentanyl and 1.5–2 mg/kg propofol and 0.1 mg/kg vecuronium was intravenously anesthetized and maintained stable hemodynamics. Routinely record all data related to anesthesia, including anesthetic dose, infusion volume, blood loss, operation time and anesthesia time, and recovery time. The whole research process uses blind method to measure, and the data is saved offline for analysis.

The evaluation of postoperative cognitive function was mainly carried out before and 3 months after the operation. The memory, learning, and attention of the patients were analyzed, respectively. In terms of executive ability and cognitive flexibility, the test indicators include digital span test, digital symbol substitution test, follow-up production test, language fluency test, and word recognition memory test [3, 17]. The evaluation of postoperative cognitive function was also blinded and analyzed by the same doctor.

### 2.3. Outcomes

In order to evaluate of POCD, we calculated the *Z* score of each indicator from the measurement results relative to the baseline data. When there are two *Z* scores in the seven test results or the composite *Z* score is greater than 1.96, the patients are divided into POCD [3]. The indicators tested include digital span test, digital symbol substitution test (DSST), follow-up production test (part A), language fluency test, and word recognition and memory test.

Among the relevant indicators of cerebral oxygen saturation detection, we calculated that rScO2 was ≥10% and ≥20% below its preoperative value. Cerebral desaturation load (CDL) is the area under the curve according to three rScO2 thresholds over time (minutes). CDL below baseline refers to the CDL below the preoperative value, CDL10 refers to the CDL load below the threshold of 10% below its preoperative value, and CDL20 refers to the CDL load below the threshold of 20% below its preoperative value. Minimum and maximum rScO2 values were defined as the lowest and highest recorded value during surgery. The prespecified main candidate predictive variable of interest was cumulative time during surgery with rScO2 ≥ 10% below its preoperative value [11, 18].

### 2.4. Statistical Analysis

The sample size was calculated to obtain a power of 0.80 at a significance level of 0.05. We sought to obtain sufficient data on early POCD (at 1–2 weeks) to discern a reduction from the previously reported 45% POCD incidence among the elderly undergoing elective joint replacement surgery to an anticipated level of about 25% which required an evaluation of 45 patients at least in each group [19].

The continuous data of the normal distribution used in this study used the mean ± standard deviation, and the continuous data of the nonnormal distribution used the median and interquartile range. The statistical difference was defined as <0.05. The *T* test was used for the comparison of normally distributed data, and the Mann-Whitney *U* test was used for nonnormal distribution. Categorical data is expressed in numbers and expressed with a 95% confidence interval and use chi-square to analysis. We use SAS version 9.3 (SAS 9.3, SAS Institute, Cary, NC) for statistics. A receiver-operating characteristic (ROC) analysis was performed to evaluate the accuracy of each parameter to distinguish between POCD and non-POCD patients.

## 3. Results

The study started to enroll patients on January 2, 2019, completed the enrollment of 90 patients on December 13, 2019, and completed the three-month follow-up in April 2020.

The 90 selected patients were divided into two groups according to the postoperative cognitive dysfunction, and 45 people were selected in each group. At the same time, during the 3-month follow-up, 19 people in the POCD group were lost to follow-up, and 15 people in the non-POCD group were lost to follow-up.  Table 1 shows the baseline data of the two groups of patients included after the operation. There is no significant difference between the two groups of patients in terms of age, education, and past history.

Table 2 shows the intraoperative conditions of the two groups of patients during the operation. There were no significant statistical differences in terms of anesthesia time, operation time, wake-up time after anesthesia, intraoperative fluid intake, and anesthetic dosage.

Table 3 shows the results of neuropsychological tests performed before and 3 months after surgery for the two groups of patients. There was no statistically significant difference in the preoperative neuropsychological evaluation. In terms of neuropsychological testing at 3 months after surgery, it was found that the POCD group had a significant decrease in correct order, and symbol digit test compared to the non-POCD group. Compared with trail making test A (s), the POCD group decreased significantly at 3 months after surgery compared with the non-POCD group.

Table 4 shows the preoperative and three-month postoperative cerebral blood oxygenation of elderly orthopedic surgery patients. We further analyzed the indicators of cerebral blood oxygenation of the two groups of patients before and 3 months after the operation. There was no significant difference in cohabitation before surgery, but at 3 months after surgery, time below preoperative rScO2, time below a 10% decrease from preoperative rScO2, CDL preoperative, minimum rScO2 value, and maximum rScO2 value were significantly higher in the non-POCD group than that in the POCD group.

Table 5 shows the correlation analysis data between the brain oxygen saturation test results and the neuropsychological function evaluation before and 3 months after the operation. We first analyzed the correlation between preoperative cerebral oxygen saturation and neuropsychological evaluation and found that there was no significant statistical difference between the two group. However, there is an obvious correlation in preoperative rScO2, time below preoperative rScO2, time below a 10% decrease from preoperative rScO2, time below a 20% decrease from preoperative rScO2, CDL preoperative, CDL20, minimum rScO2 value, and maximum rScO2 values at 3 months postoperatively.

**Figure 1:**
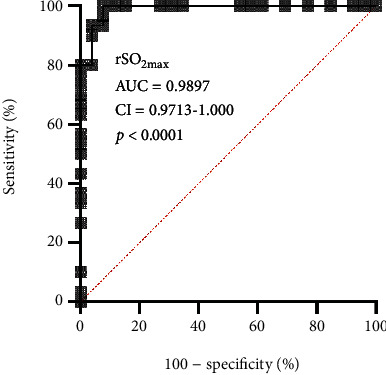
A multilogistic regression analysis found that rSO 2%max is an independent predictor of POCD.

Besides, there is an obvious correlation between digit span test and preoperative rScO2. Correct order also correlated with mean rScO2 during surgery and time below preoperative rScO2. Reverse order correlated with minimum rScO2 value and maximum rScO2 value. Trail making test A correlated with preoperative rScO2. Verbal fluency test verbal correlated with preoperative rScO2, minimum rScO2 value, and maximum rScO2 value. Word recognition memory test correlated with minimum rScO2 value and maximum rScO2 value. At the same time, we also carried out a multilogistic regression analysis and found that rSO 2%max is an independent predictor of POCD (Figure 1).

## 4. Discussion

In our study, we analyzed the relationship between inoperative cerebral oxygen and degree testing and postoperative cognitive dysfunction and found a clear correlation between the effects of inoperative cerebral oxygenation testing three months after surgery and postoperative cognitive dysfunction.

POCD is a central nervous system-related complication after surgery, manifested as confusion, anxiety, changes in personality and behavior, and memory impairment [20] which can decrease in memory and concentration after surgery. Besides, there are many reasons related with POCD, such as the type of surgery, the level of cognitive function of the patients before the operation, the related medication history, the persistent hypotension after the operation, and the persistent hypoxia after the operation [21]. The central nervous system gradually degenerates with the increase of age. POCD also can delay patient's recovery time, prolong the hospital stay, and increase the economic pressure of the patient [22]. Relevant studies have shown that the incidence of POCD in elderly patients within 1 week after noncardiac surgery is about 29.6%, of which the incidence of POCD in general surgery abdominal surgery is 38.0%, and the incidence of POCD in orthopedic surgery is 25.0% [3, 4, 23–29]. It can be seen that the incidence of POCD is relatively high, and it usually occurs within 1 week after surgery. Therefore, it is important to find a way to predict postoperative cognitive dysfunction in patients at an early stage.

The occurrence of POCD in the elderly is mostly related to intraoperative fluctuation of blood pressure and cerebral hypoxia [22]. The use of drug during anesthesia process also significantly affects the development of POCD. Glumac et al. also found that the prophylactic administration of dexamethasone seems to be useful to prevent POCD development following cardiac surgery [30]. Insomnia will lead to increased aldosterone secretion in the body and increased blood pressure [23, 24, 31]. Besides, the central nervous system of the brain is highly sensitive to hypoxia, and hypoxia will also affect the functional state of the brain. Moderate hypoxia can cause a decline in the function of brain's cholinergic nervous system and affect the release of central neurotransmitters in the brain, thus damaging brain's function. Therefore, intraoperative low cerebral oxygen saturation or cerebral blood perfusion is one of the causes of POCD in patients undergoing surgery [32]. Therefore, this study further analyzes the relationship between cerebral oxygen saturation and postoperative cognitive function.

The rSO2 monitoring technology was first proposed by Jobsis in 1977. The principle is to use the difference in absorption of near-infrared light of different wavelengths by human tissues. rSO2 monitoring is mainly used to reflect the oxygenation changes of the body tissues under hypotension, hypovolemia, embolism, shock-induced ischemia, internal environmental changes, etc., due to its practical effect and high accuracy; it is noninvasive and insensitive to the body. With advantages such as temperature influence, clinical applications are increasing day by day [33]. As a noninvasive monitoring method to effectively assess the balance of cerebral oxygen supply and demand and changes in cerebral blood flow, rSO2 monitoring has important clinical value for the diagnosis and prevention of patients' perioperative adverse reactions [34]. Salazar et al. analyzed the correlation between intraoperative rSO2 and postoperative cognitive function in 125 patients over 65 years of age who underwent total knee arthroplasty under subarachnoid block anesthesia and found that 21 patients (16.8%) were postoperative memory decreased, 3 patients (2.4%) had a drop in the Weishi Memory Scale score, 33 patients (26.4%) had psychological symptoms, and the left and right rSO2 values of patients with memory loss appeared asymmetry [15]. Trafidlo et al. randomized 43 patients who underwent prone lumbar spine surgery (including laminectomy, hemilaminectomy, and lumbar discectomy). A group of patients was monitored in real-time rSO2 during the operation and intervened when the value fluctuates. Measures (*n* = 13, 30.2%), the other group did not conduct rSO2 monitoring (*n* = 30, 69.8%) [35]. The results showed that the incidence of POCD in the monitoring group was lower, and intraoperative rSO2 monitoring helped to reduce the incidence of postoperative cognitive decline in patients with prone lumbar spine surgery. Lin et al. found that the maximum decrease percentage of rSO2 during total hip arthroplasty can be used as an important predictor of POCD, and the maximum decrease percentage of rSO2 over 11% can be used as a potential predictor of neurocognitive impairment [14]. However, some scholars have put forward different opinions. Nakao et al. continuously monitored rSO2 during the operation of 50 patients undergoing shoulder surgery on the beach chair position and analyzed the correlation with postoperative cognitive function changes [36]. In this study, each patient underwent neuropsychological status assessment on the day of surgery, the first day before discharge, and the third day after discharge. The content included the measurement of immediate memory, vision, spatial structure, language, attention, and delayed memory. A reduction of 20% or more in rSO2 at any time during the operation was defined as a brain desaturation event. The results showed that although the position of the beach chair during the operation may cause insufficient cerebral perfusion and 18% of patients had brain desaturation during the operation, there was no statistical significance between the occurrence of intraoperative brain desaturation and POCD. The reason for Salazar's different conclusions may be related to the early detection of brain desaturation during the operation and the prompt and rapid intervention to reverse brain desaturation and because the evaluation of patient's postoperative cognitive function is only traced to the 3rd day after discharge. Therefore, whether intraoperative rSO2 will affect long-term cognitive function remains to be confirmed by further research.

In this study, we found that there was a clear correlation between cognitive dysfunction and cerebral oxygen saturation in patients 3 months after surgery, which provided important reference for the prevention and treatment of cognitive dysfunction after orthopedic surgery in future elderly patients.

### 4.1. Study Limitations

Limitations of this study include the following points. Firstly, the sample size of this study is small, and the lack of long-term follow-up results. At the same time, follow-up time can be up to six months, which leads to the loss of follow-up. In addition, the results of this study were lacking in the assessment of blood oxygen saturation and cognitive impairment in patients discharged from the hospital after surgery. Secondly, in this study, the detection of cerebral oxygen saturation in surgery is just one method that may lack more help in the treatment of postoperative cognitive impairment. Therefore, it may be necessary to analyze further treatment methods in future studies. Third, the last study is not a randomized controlled study. Although the patients are grouped according to the cognitive dysfunction of the postoperative patients, the intraoperative blood oxygen saturation is collected blindly, so as to minimize the occurrence of deviation. Finally, the classification of orthopedic surgery in this study was mainly divided into joint surgery and spine surgery, and trauma surgery was not included in the study. At the same time, there is no distinction between spinal surgery and joint surgery, which may be further analyzed in the future when the sample size is expanded.

## Figures and Tables

**Table 1:** Characteristics of the patients.

Patient characteristic	All patient (90)	No-POCD (45)	POCD (45)	*P* value
Age (year)	65.3 (59.2; 75.2)	64.2 (52.3; 71.2)	65.8 (53.6; 77.2)	0.581
BMI	27.2 (2.2)	27.5 (3.1)	27.5 (2.8)	0.612
*Sex(%)*				
Male, *n* (%)	46	21	25	0.592
Female, *n* (%)	44	24	20	0.351
*ASA physical status (%)*				
II	50.5	43	58	0.154
III	49.47	57	42	0.251
*Educational level (%)*				
Less than high school	18	15	21	0.514
High school	60.5	59	62	0.261
More than high school	21.5	26	17	0.241
*Comorbidity, before operation,n(%)*				
Hypertension	57	56	58	0.147
Diabetes	11	10	12	0.251
Smoking history (%)	12	15	9	0.368
Surgical history (%)	19	20	18	0.215
*Surgery*				
Joint surgery (%)	54.5	58	51	0.251
Spine surgery (%)	45.5	42	49	0.258

Data are presented as mean ± SD, or median (5th–95th percentile), or percentage of all patients. POCD: postoperative cognitive dysfunction; BMI: body mass index; ASA: American Society of Anesthesiologists.

**Table 2:** Intraoperative data between the two group.

Intraoperative data	No-POCD (45)	POCD (45)	*P* value
Duration of anesthesia (min)	146.02 ± 25.15	152 ± 31.22	0.351
Duration of surgery (min)	108.61 ± 36.15	118.02 ± 12.56	0.151
Recovery time (min)	41.02 ± 12.02	39.05 ± 11.92	0.201
Fluid replacement (ml)	2481 ± 231.02	2591 ± 217.05	0.398
Blood loss (ml)	605.52 ± 23.20	632.18 ± 15.25	0.521
Fentanyl (mg)	0.41 ± 0.01	0.39 ± 0.02	0.151
Vecuronium (mg)	11.02 ± 2.11	10.08 ± 1.25	0.092

Data are presented as mean ± SD, or median (5th–95th percentile), or percentage of all patients. POCD: postoperative cognitive dysfunction.

**Table 3:** Neuropsychological test results for geriatric orthopedic surgery.

	Cognitive dysfunction at baseline		*P* value	Cognitive dysfunction at 3 months		*P* value
	No-POCD (45)	POCD (45)		No-POCD (30)	POCD (26)	
Correct order	8.26 ± 0.86	8.00 ± 1.15	0.084	8.31 ± 0.94	7.02 ± 2.15	0.045
Reverse order	4.02 ± 2.02	4.61 ± 0.25	0.154	4.53 ± 1.02	q	0.514
Symbol digit test	33.61 ± 12.05	31.05 ± 6.25	0.123	32.61 ± 5.94	24.58 ± 4.61	0.011
Trail making test A (s)	18.62 ± 8.25	19.36 ± 5.61	0.358	18.92 ± 3.64	21.59 ± 4.61	0.032
Verbal fluency test	16.89 ± 3.15	13.05 ± 8.2	0.261	16.02 ± 3.25	11.25 ± 6.32	0.325
Word recognition memory tests	1.02 ± 0.25	1.26 ± 0.23	0.154	1.15 ± 0.51	1.48 ± 0.59	0.114

Data are presented as mean ± SD. POCD: postoperative cognitive dysfunction.

**Table 4:** Regional cerebral oxygen saturation value for geriatric orthopedic surgery.

Patient characteristic	Cognitive dysfunction at baseline		Difference (95% CI)	*P* value	Cognitive dysfunction at 3 months		Difference (95% CI)	*P* value
	No-POCD (45)	POCD (45)			No-POCD (30)	POCD (26)		
Preoperative rScO2	67.81 ± 2.36	65.02 ± 1.21	0.8 (-2.0; 3.2)	0.053	61.25 ± 1.06	66.25 ± 2.36	0.8 (-2.0; 3.2)	0.625
Mean rScO2 during surgery	66.32 ± 2.61	64.02 ± 1.12	1.2 (-1.2; 2.3)	0.251	60.36 ± 2.64	67.25 ± 1.25	3.2 (-6.3; 2.3)	0.514
Time below preoperative rScO2 (min)	58.42 ± 2.36	62.05 ± 0.26	2.8 (-5.3; 2.6)	0.125	68.25 ± 1.02	52.61 ± 3.25	3.6 (-6.5; 3.1)	0.015
Time below a 10% decrease from preoperative rScO2 (min)	6.02 ± 1.12	15.62 ± 2.36	5.9 (-5.8; 2.6)	0.325	18.25 ± 1.51	6.02 ± 0.95	2.3 (-1.1; 2.0)	0.011
Time below a 20% decrease from preoperative rScO2 (min)	0.23 ± 0.05	0.21 ± 0.02	0.0 (-0.2; 0.1)	0.125	0.21 ± 0.01	0.11 ± 0.02	0.1 (-0.2; 0.3)	0.09
CDL preoperative (% × min)	198.85 ± 12.09	405.36 ± 12.05	53.6 (-53.1; 26.3)	0.102	401.25 ± 3.61	185.02 ± 2.31	61.25 (-73.61; 56.36)	0.025
CDL10 (% × min)	10.25 ± 2.23	28.61 ± 1.02	6.5 (-5.9; 6.2)	0.089	31.25 ± 3.61	12.25 ± 2.36	8.61 (-6.3; 7.25)	0.364
CDL20 (% × min)	0.02 ± 0.01	0.03 ± 0.01	0.0 (-0.3; 0.5)	0.521	0.00 ± 0.02	0.01 ± 0.01	0.0 (-0.2; 0.5)	0.085
Minimum rScO2 value	55.26 ± 2.36	51.25 ± 1.15	2.3 (-1.2; 2.6)	0.261	63.68 ± 1.25	55.26 ± 3.61	3.6 (-4.5; 5.1)	0.02
Maximum rScO2 value	82.09 ± 5.26	80.61 ± 2.03	5.3 (-3.6; 2.3)	0.251	89.36 ± 6.32	83.61 ± 5.61	6.1 (-5.1; 6.1)	0.01

Regional cerebral oxygen saturation (rScO2).CDL: cerebral desaturation load (area under these thresholds). Data are presented as mean ± SD, or median (5th–95th percentile), or percentage of all patients. POCD: postoperative cognitive dysfunction.

**Table 5:** Correlation between regional cerebral oxygen saturation (rScO2) variables and selected cognitive test variable elements for patients with postoperative cognitive dysfunction (POCD) at baseline and 3 months after surgery.

	Digit span test		Correct order		Reverse order		Symbol digit test		Trail making test A		Verbal fluency test		Word recognition memory tests	
	*R*	*P* value	*R*	*P* value	*R*	*P* value	*R*	*P* value	*R*	*P* value	*R*	*P* value	*R*	*P* value
*Correlation analysis in baseline*														
Preoperative rScO2	-0.05	0.51	-0.02	0.45	-0.11	0.61	-0.02	0.52	0.02	0.61	0.01	0.52	0.02	0.25
Mean rScO2 during surgery	0.06	0.62	0.03	0.51	0.25	0.25	0.03	0.13	0.01	0.17	0.02	0.25	0.03	0.61
Time below preoperative rScO2 (min)	0.01	0.35	0.01	0.41	-0.02	0.15	0.02	0.36	0.02	0.25	0.03	0.36	-0.05	0.35
Time below a 10% decrease from preoperative rScO2 (min)	0.02	0.64	0.02	0.25	0.03	0.62	0.01	0.21	-0.01	0.36	0.02	0.33	0.02	0.36
Time below a 20% decrease from preoperative rScO2 (min)	0.01	0.81	0.03	0.32	0.05	0.25	0.02	0.36	0.02	0.18	-0.01	0.22	0.01	0.25
CDL preoperative (% × min)	-0.03	0.26	-0.03	0.36	0.02	0.44	0.02	0.25	0.03	0.09	0.05	0.35	0.02	0.36
CDL10 (% × min)	0.02	0.61	0.01	0.51	0.03	0.36	-0.05	0.26	0.06	0.18	0.01	0.12	0.05	0.36
CDL20 (% × min)	0.03	0.36	0.02	0.25	-0.01	0.25	0.02	0.65	0.05	0.24	0.02	0.36	0.01	0.31
Minimum rScO2 value	-0.02	0.64	0.02	0.61	0.02	0.68	0.01	0.89	0.02	0.21	0.03	0.25	0.02	0.81
Maximum rScO2 value	0.05	0.25	0.03	0.32	0.02	0.25	0.03	0.91	-0.05	0.22	0.01	0.39	-0.05	0.64
*Correlation analysis in 3 months*														
Preoperative rScO2	0.23	0.02	0.05	0.52	0.05	0.61	0.05	0.62	0.02	0.02	0.62	0.02	0.26	0.02
Mean rScO2 during surgery	0.21	0.61	0.61	0.01	-0.06	0.35	0.36	0.36	0.03	0.36	0.36	0.36	0.32	0.62
Time below preoperative rScO2 (min)	0.02	0.35	0.02	0.02	0.02	0.33	-0.25	0.35	0.01	0.36	0.02	0.35	0.12	0.36
Time below a 10% decrease from preoperative rScO2 (min)	0.03	0.61	0.36	0.23	0.01	0.36	0.61	0.25	0.03	0.15	0.03	0.36	0.36	0.15
Time below a 20% decrease from preoperative rScO2 (min)	0.01	0.81	-0.05	0.51	-0.06	0.15	0.36	0.36	-0.02	0.36	0.25	0.02	0.25	0.25
CDL preoperative (% × min)	0.02	0.36	0.02	0.36	0.21	0.25	0.32	0.15	0.03	0.35	-0.03	0.36	-0.05	0.36
CDL10 (% × min)	-0.36	0.35	0.31	0.15	0.02	0.48	0.25	0.58	0.05	0.25	0.36	0.25	0.03	0.25
CDL20 (% × min)	0.21	0.25	0.05	0.52	0.36	0.36	0.25	0.25	0.61	0.36	0.25	0.32	0.02	0.15
Minimum rScO2 value	0.06	0.23	0.06	0.33	0.02	0.02	-0.25	0.61	0.25	0.25	0.36	0.02	0.32	0.02
Maximum rScO2 value	0.02	0.02	0.04	0.11	0.03	0.01	0.03	0.25	0.36	0.25	-0.03	0.01	0.22	0.03

## Data Availability

All data generated or analysed during this study are included in this published article.

## Abbreviations

POCD: Postoperative cognitive dysfunction
DSST: Digital symbol substitution test
CDL: Cerebral desaturation load.
